# Deep‐Intronic Variant in *RUNX2* Causing Pseudo‐Exon Inclusion in a Family With Cleidocranial Dysplasia

**DOI:** 10.1111/cge.70158

**Published:** 2026-03-08

**Authors:** Dorothea Stojanovic, Dorota Garczarczyk‐Asim, Julia Vodopiutz, Andreas R. Janecke

**Affiliations:** ^1^ Department of Pediatrics I Medical University of Innsbruck Innsbruck Austria; ^2^ Department of Paediatrics and Adolescent Medicine, Division of Neonatology, Intensive Care Medicine and Neuropediatrics, Comprehensive Center for Paediatrics Medical University of Vienna Vienna Austria; ^3^ ERN BOND Full Member and Vienna Bone and Growth Center Vienna Austria; ^4^ Institute of Human Genetics Medical University of Innsbruck Innsbruck Austria

**Keywords:** cleidocranial dysplasia, long‐read sequencing, pseudo‐exon inclusion, RUNX2

## Abstract

A deep‐intronic single nucleotide variant in *RUNX2* causes the characteristic clinical features of cleidocranial dysplasia (CCD) in a family via pseudo‐exon inclusion into the mRNA. The pseudo‐exon contains a premature stop codon and triggers mRNA decay, which results in *RUNX2* haploinsufficiency, the known disease mechanism.

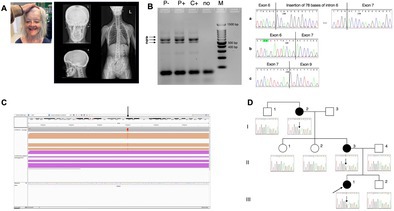

Skeletal dysplasias represent a large group of clinically and genetically heterogenous disorders affecting bone development. Cleidocranial dysplasia (CCD) is a rare congenital, autosomal‐dominantly inherited disorder with a wide range of symptoms and varying degrees of severity. CCD is most commonly caused by heterozygous loss‐of‐function variants in the transcription factor *RUNX2*, and rarely by heterozygous loss‐of‐function variants in *CBFB*, encoding the regulatory beta subunit of a core‐binding transcription factor upstream of *RUNX2* [1].

Exome sequencing was performed in three individuals with CCD from a family (Figure 1A). Written informed consent for molecular studies and publication of data and photographs was obtained from the patients. This approach was approved by the ethics committee of the Medical University of Innsbruck. However, no pathogenic variants were present in the coding regions of *RUNX2* and *CBFB* despite good coverage (100% of targeted bases covered more than 20 times).

**FIGURE 1:**
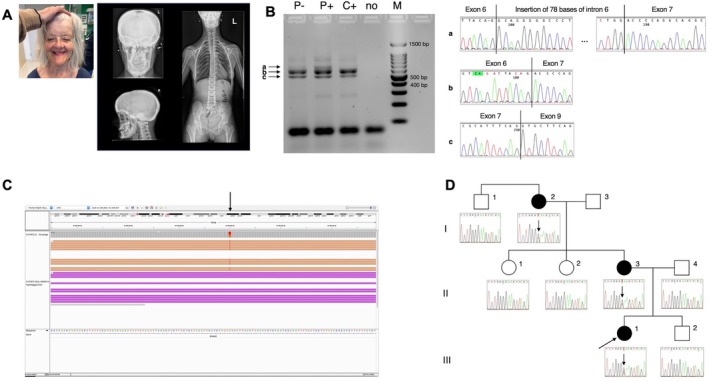
A deep‐intronic *RUNX2* variant causing CCD. The proband (III/1) was referred to establish a clinical and molecular diagnosis of CCD, and family members were recruited for support. (A) Clinical photographs of an adult patient (I/2) presenting with facial dysmorphism including open anterior fontanel, frontal bossing, hypertelorism and midfacial hypoplasia. Skeletal radiographs show parietal bossing, open fontanel, midface hypoplasia, hypoplastic clavicles, narrow thorax and wide pubic symphysis in individual II/3. (B) PCR amplification of exons 6–9 of *RUNX2* cDNA reveals an aberrant band that harbours 78 bases of intron 6 sequence in affected individual II/3. Two further bands present in this patient and control correspond to canonical transcript ENST00000647337.2 and to splice‐variant ENST00000371432.7. P+ and P‐, patient's mRNA extracted with (+) and without (−) protein synthesis inhibition in fibroblast cultures 6 h prior to cell harvesting with puromycin (to a final concentration of 200 μg/mL); C, control fibroblast mRNA; M, molecular weight marker. (C) Haplophased long‐read genome sequencing displaying deep‐intronic variant NC_000006.12:g.45509900G>T (arrow) in *RUNX2* in individual I/2, and (D) family tree showing variant segregation with CCD as determined by Sanger sequencing of part of RUNX2 intron 6 in genomic DNA from all displayed family members.

To identify the CCD‐causing variant in this family, the canonical *RUNX2* transcript was amplified in fibroblast‐derived cDNA from an affected individual and controls in four overlapping fragments and sequenced. In addition to a wild‐type transcript, the patient produced an aberrant transcript that contained 78 bases of intron 6 sequence interspersed between exons 6 and 7. The aberrant transcript (NM001024630.4:c.859_860ins78) contains a premature stop‐gain (p.Asp287Glyfs*3), and we show that it undergoes nonsense‐mediated mRNA decay (Figure 1B). We subsequently performed PacBio Revio long‐read genome sequencing in this patient; we identified a single rare *RUNX2* variant within intron 6, which was absent from the gnomAD v4.1.0 population database (NC_000006.12:g.45509900G>T, Figure 1C), and segregates with CCD in the family (Figure 1D). Splice prediction using SpliceAIlookup (https://spliceailookup.broadinstitute.org) indicated that this variant resulted in the inclusion of a pseudo‐exon of 78 bp, exactly corresponding to the aberrant cDNA transcript observed.

Collectively, we demonstrated haploinsufficiency as the typical mechanism to be responsible for CCD in this family. To date, there are 151 known variants in *RUNX2* that are classified as pathogenic or likely pathogenic (https://www.ncbi.nlm.nih.gov/clinvar), and the majority are exonic premature stop‐gain variants. To the best of our knowledge, we demonstrate here the first instance of pseudo‐exon inclusion resulting from a deep‐intronic variant in *RUNX2* in CCD.

Pseudo‐exon inclusion has emerged as an important pathogenic mechanism in an increasing number of monogenetic disorders. Pseudo‐exon inclusion results from intronic variants that activate cryptic splice sites [2]. This mechanism may account for a significant percentage of patients, as demonstrated particularly in neurofibromatosis type 1 [3] and in the genetically heterogeneous group of retinal dystrophies [4]. Genome sequencing has proven to identify such clinically relevant variants which are not detected by standard approaches [5]. The analysis of splice defects can enhance genetic diagnoses and has become crucial with the rise of genetic therapies and a shift toward personalized medicine, especially as the use of antisense oligonucleotides has already shown promising results.

## Conflicts of Interest

The authors declare no conflicts of interest.

## Data Availability

The data that support the findings of this study are available from Patients. Restrictions apply to the availability of these data, which were used under license for this study. Data are available from the author(s) with the permission of Patients.

## References

[1] T. Beyltjens , E. Boudin , N. Revencu , et al., “Heterozygous Pathogenic Variants Involving CBFB Cause a New Skeletal Disorder Resembling Cleidocranial Dysplasia,” Journal of Medical Genetics 60, no. 5 (2023): 498–504. 36241386 10.1136/jmg-2022-108739 PMC10176335

[2] R. Vaz‐Drago , N. Custodio , and M. Carmo‐Fonseca , “Deep Intronic Mutations and Human Disease,” Human Genetics 136, no. 9 (2017): 1093–1111. 28497172 10.1007/s00439-017-1809-4

[3] M. Koczkowska , Y. Chen , J. Xie , et al., “Analysis of 200 Unrelated Individuals With a Constitutional NF1 Deep Intronic Pathogenic Variant Reveals That Variants Flanking the Alternatively Spliced NF1 Exon 31 [23a] Cause a Classical Neurofibromatosis Type 1 Phenotype While Altering Predominantly NF1 Isoform Type II,” Human Genetics 142, no. 7 (2023): 849–861. 37186028 10.1007/s00439-023-02555-z PMC10329576

[4] J. Reurink , N. Weisschuh , A. Garanto , et al., “Whole Genome Sequencing for USH2A‐Associated Disease Reveals Several Pathogenic Deep‐Intronic Variants That Are Amenable to Splice Correction,” HGG Adv 4, no. 2 (2023): 100181. 36785559 10.1016/j.xhgg.2023.100181 PMC9918427

[5] X. Liu , F. Hu , D. Zhang , et al., “Whole Genome Sequencing Enables New Genetic Diagnosis for Inherited Retinal Diseases by Identifying Pathogenic Variants,” NPJ Genomic Medicine 9, no. 1 (2024): 6. 38245557 10.1038/s41525-024-00391-2 PMC10799956

